# Cytosine Methylation Changes the Preferred Cis-Regulatory Configuration of Arabidopsis WUSCHEL-Related Homeobox 14

**DOI:** 10.3390/ijms26020763

**Published:** 2025-01-17

**Authors:** Dingkun Jiang, Xinfeng Zhang, Lin Luo, Tian Li, Hao Chen, Nana Ma, Lufeng Fu, Peng Tian, Fei Mao, Peitao Lü, Honghong Guo, Fangjie Zhu

**Affiliations:** 1 College of Life Science, Haixia Institute of Science and Technology, National Engineering Research Center of JUNCAO, Fujian Agriculture and Forestry University, Fuzhou 350002, China; jiangdk0722@163.com (D.J.);; 2 College of Horticulture, Fujian Agriculture and Forestry University, Fuzhou 350002, China; 3 National Key Laboratory for Tropical Crop Breeding, Institute of Tropical Bioscience and Biotechnology, Chinese Academy of Tropical Agricultural Sciences, Sanya 572024, China

**Keywords:** WUSCHEL-related homeobox 14, DNA binding specificity, SELEX, DNA methylation, transcription factor, dimeric spacing

## Abstract

The Arabidopsis transcription factor WUSCHEL-related homeobox 14 (AtWOX14) plays versatile roles in plant growth and development. However, its biochemical specificity of DNA binding, its genome-wide regulatory targets, and how these are affected by DNA methylation remain uncharacterized. To clarify the biochemistry underlying the regulatory function of AtWOX14, using the recently developed 5mC-incorporation strategy, this study performed SELEX and DAP-seq for AtWOX14 both in the presence and absence of cytosine methylation, systematically curated 65 motif models and identified 51,039 genomic binding sites for AtWOX14, and examined how 5mC affects DNA binding of AtWOX14 through bioinformatic analyses. Overall, 5mC represses the DNA binding of AtWOX14 monomers but facilitates the binding of its dimers, and the methylation effect on a cytosine’s affinity to AtWOX14 is position-dependent. Notably, we found that the most preferred homodimeric configuration of AtWOX14 has changed from ER1 to ER0 upon methylation. This change has the potential to rewire the regulatory network downstream of AtWOX14, as suggested by the GO analyses and the strength changes in the DAP-seq peaks upon methylation. Therefore, this work comprehensively illustrates the specificity and targets of AtWOX14 and reports a previously unrecognized effect of DNA methylation on transcription factor binding.

## 1. Introduction

The homeobox (HB) superfamily, defined by a conserved homeodomain (HD) of 60–66 amino acids, is widely distributed across plants and animals [1]. HB superfamily members serve as key regulators of growth and development. Plant HB superfamily TFs are involved in various processes, such as embryo patterning [2], organ development (root, shoot, floral meristems) [3], vascular development, and stress responses [4,5,6,7]. Although the HB superfamily is shared in eukaryotes, the HB TFs belonging to the WUSCHEL (WOX) family are plant-specific. WOX TFs are phylogenetically categorized into the modern, intermediate, and ancient clades based on evolutional evidence and species distributions [8]. The Arabidopsis TF—AtWOX14 (WUSCHEL-related homeobox 14) is a member of the ancient clade. AtWOX14 plays critical roles in diverse developmental processes, including bud regeneration in callus [9], lateral root and stamen development [10], vascular cell differentiation [11], lignification [12], and the accumulation of bioactive gibberellin in the stem of inflorescence [13].

To regulate the expression of target genes, transcription factors (TFs) selectively bind to specific DNA sequences. This binding process can be influenced by chromatin accessibility [14,15], histone modifications [16,17], TF-TF interaction (cooperative or competitive) [18,19], and DNA methylation [20,21]. DNA methylation typically inhibits TF binding, for example, plant WRKY family TFs show reduced DNA-binding affinities upon cytosine methylation [22]. However, some TFs can enhance their binding affinity to methylated DNA [23,24,25]. For example, a comprehensive survey of 542 TFs reported that TFs critical for early embryonic development (e.g., POU5F1 and HOXB13) preferentially bind methylated DNA, highlighting the dual role of methylation in modulating TF-DNA interactions [23]. TFs frequently bind DNA as dimers; for instance, ARF forms homodimers upon binding [26], and bZIP10 and bZIP53 can form heterodimers [18]. While the previous studies systematically addressed the impact of methylation on the nucleotide specificity of TFs [23], limited insights exist regarding how 5mC affects the configuration of dimeric binding of TFs.

Given the critical roles of AtWOX14 in plant growth and development, this study aims to thoroughly clarify its DNA-binding specificity and regulatory targets and how DNA methylation influences these. Both in the absence and presence of methylation, altogether we curated 4 monomeric and 63 dimeric binding models for AtWOX14 and validated the ER0/1 dimeric binding model with EMSA. Methylation has globally changed the binding specificity of AtWOX14, not only the sequence specificity but, notably, also the configurational specificity (the spacing between the monomers) of dimerization. The specificity changes upon methylation also dictate the binding strength of AtWOX14 to its genomic targets—either being enhanced or inhibited depending on the sequence of the cis-regulatory elements (CREs) of AtWOX14, suggesting that the methylation effect on AtWOX14′s regulatory activity can be complex.

For AtWOX14, here we use SELEX to profile the biochemical specificity and DAP-seq to identify the genome-wide regulatory targets. This is because the randomness and complexity of the SELEX input library ensures the unbiased and sensitive discovery of a TF’s binding models [27,28], while DAP-seq uses genomic DNA to directly interrogate the binding sites and target genes of the TF [25]. These two methods complement each other and are preferably combined. Also, in contrast to animals whereby the majority of methylation happens on the CG dinucleotides, in plants, methylation can occur in all of the CG, CHG, and CHH contexts [29]. Taking this into consideration, in this study we replaced all Cs in the SELEX/DAP-seq library with 5mCs when examining the methylation effects, by using a recently developed protocol [30].

## 2. Results

### 2.1. Methylation Inhibits Monomeric Binding but Enhances Dimeric Binding of AtWOX14

**Figure 1:**
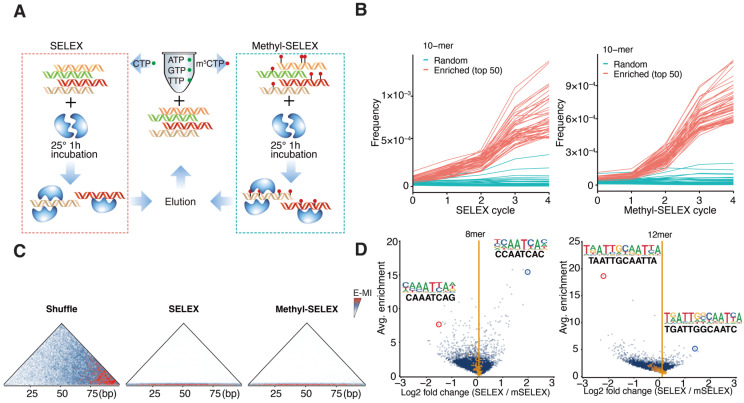
The binding specificity of WOX14 in the presence and absence of methylation. (**A**) Schematic representation of the SELEX and Methyl-SELEX workflow. (**B**) The frequencies of subsequence (10-mers) in each cycle of WOX14 SELEX. In addition to the bound sequences (top 50 enriched sequences, red), 50 randomly selected sequences (blue) were also visualized, (**C**) E-MI analyses of the SELEX libraries show that the signal near the hypotenuse of the triangle becomes stronger than elsewhere, a pattern indicating the enrichment of TF signals, (**D**) Enrichment comparison of 8-mers and 12-mers between SELEX and Methyl-SELEX libraries.

To unravel the DNA-binding specificity of AtWOX14 to unmodified DNA, we first performed SELEX without methylation (Figure 1A). Recombinant AtWOX14 protein was incubated with a 101-bp DNA library containing a randomized region. The ligands with high affinities to AtWOX14 were then purified together with AtWOX14 and amplified for the next cycle. To assess the impact of methylation on the specificity of AtWOX14, the SELEX process was also modified to incorporate 5mCs instead of Cs during the PCR amplification [30], generating a fully methylated DNA library in each cycle (Figure 1A). After five cycles, the enriched DNA pools were sequenced and analyzed.

We first confirmed that SELEX enriched binding signals of AtWOX14. For example, specific 10-mer subsequences were enriched over SELEX cycles (Figure 1B). Such enrichment is exponential because a straight line is observed after taking the logarithm (Figure S1). Enrichment-based mutual information (E-MI) analysis [31,32] has also validated the presence of TF-binding signals in the enriched library (Figure 1C).

We next investigated the impact of methylation on the preferred kmers of AtWOX14 (Figure 1D). Interestingly, when we compared all 8-mer subsequences between the methylated and normal libraries, the most abundant 8-mers were more enriched in the SELEX library (Figure 1D, left). In contrast, when all 12-mer subsequences were compared, the most abundant 12-mers were enriched in the Methyl-SELEX library (Figure 1D, right). We further performed motif discovery using 8-mers and 12-mers as seeds (using Autoseed [33]) and found that 8-mers correspond to monomeric AtWOX14 motifs, while 12-mers correspond to dimeric motifs. Therefore, it can be concluded that methylation inhibits the monomeric binding of AtWOX14, while enhancing its dimeric binding.

### 2.2. Methylation Changes the Preferred Homodimeric Configuration of AtWOX14

**Figure 2:**
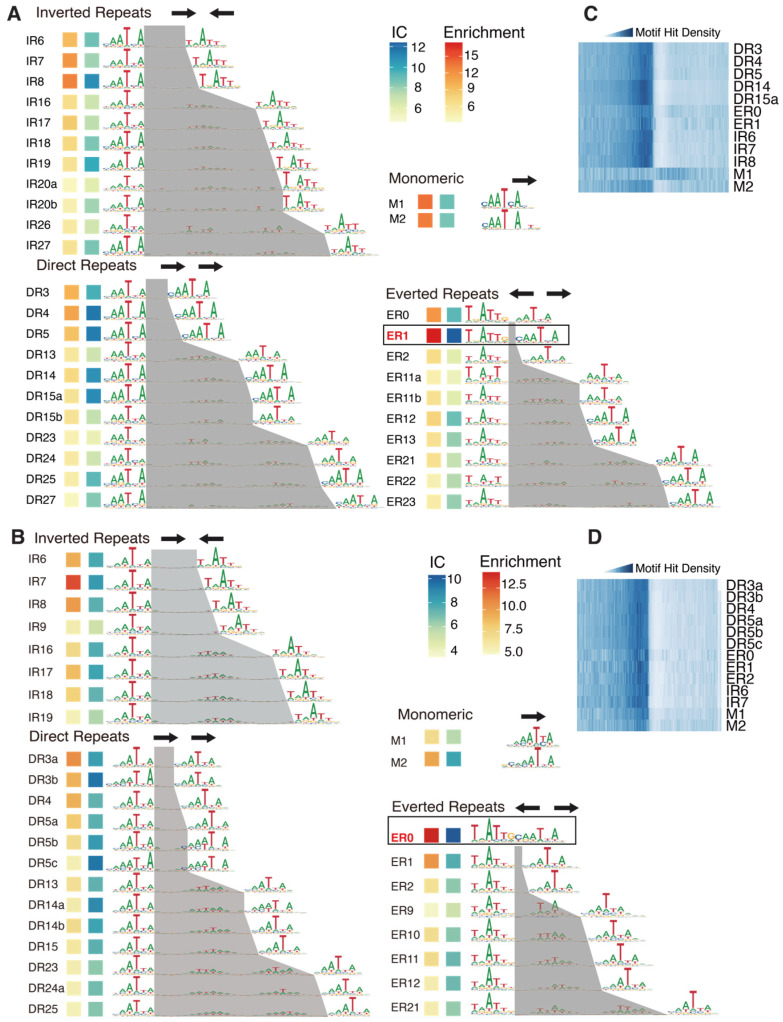
The binding models of WOX14 in the presence and absence of methylation. De novo discovered motifs in the SELEX (**A**) and Methyl-SELEX (**B**) libraries are shown, with their information content (blue squares) and enrichment (red squares) indicated to the left. By defining the half-site as “AATYA”, the spacing regions of the dimeric motifs are colored gray. Arrows on top of the motif groups indicate the relative orientations of the monomers in the dimeric binding. Distributions of the discovered motifs (mean IC > 0.3) around TSSs of *A. thaliana* genes were evaluated by the density of motif matches and visualized for both the SELEX motifs (**C**) and Methyl-SELEX motifs (**D**).

While methylation is known to affect TFs’ nucleotide specificity and affinity [23,25], how the incorporation of 5mC affects TFs’ dimeric configuration is yet to be explored. We noticed that, depending on methylation, the most enriched dimeric motifs exhibited different configurations regarding the spacings of the half-sites (Figure 1D, right). Therefore, we next de novo identified all dimeric (and monomeric) binding motifs of WOX14 (Figure 2A,B) with the reported method [33] and compared their enrichments between the SELEX and the Methyl-SELEX libraries.

The identified dimeric motifs covered all possible relative orientations of monomers (direct repeats: DRs, inverted repeats: IRs, and everted repeats: ERs). By defining the half-site as “YAATYA”, we can calculate the spacing between the half-sites and name each dimeric motif by combining the relative orientation and the spacing (the name is labeled to the left of each motif, Figure 2A,B).

From the normal SELEX library, we identified 34 motif models, including 2 monomeric motifs and 32 homodimeric motifs (Figure 2A). In general, the dimeric motifs with narrower spacings displayed greater enrichments and higher information content (IC) (Figure 2A). The ER1 motif exhibited the highest IC and enrichment, followed by ER0, DR3-5, and IR6-8 (Figure 2A). An important signature of the functional TF motifs is that they are enriched around transcription start sites (TSSs). Accordingly, we found that the identified high-IC dimeric motifs were all enriched upstream of TSSs (Figure 2C). However, we observed that the dimeric models showed higher enrichments at TSSs compared to the monomeric models. This is consistent with the stronger binding signals of dimeric models in SELEX (Figure 2A), and potentially also because the higher transactivating activity of the dimeric CREs is more strongly selected during evolutional history. To date, the reported motifs (e.g., in public databases [34,35,36]) of WOX TFs have been mainly monomeric. Here, we show that the dimeric motifs are more enriched in SELEX and around the TSSs, revealing that WOX14 has a strong preference for binding as homodimers.

In the presence of methylation, we identified 2 monomeric motifs and 29 dimeric motifs, including 8 IR, 13 DR, and 8 ER motifs (Figure 2B). Consistent with the SELEX results (Figure 2A), dimeric motifs show higher enrichment and IC compared to the monomeric motifs, further supporting the preference of WOX14 to bind as a dimer. The high-IC motifs are also enriched around TSSs (Figure 2D). Interestingly, the most enriched dimeric motif has switched from ER0 to ER1 (Figure 2B). The motif-based enrichment analysis here is also consistent with the 12-mer-based analysis (Figure 1D, right), confirming the methylation effect on the homodimeric configuration of AtWOX14.

### 2.3. The Targets of ER0 and ER1 Are of Different Physiological Functions

**Figure 3:**
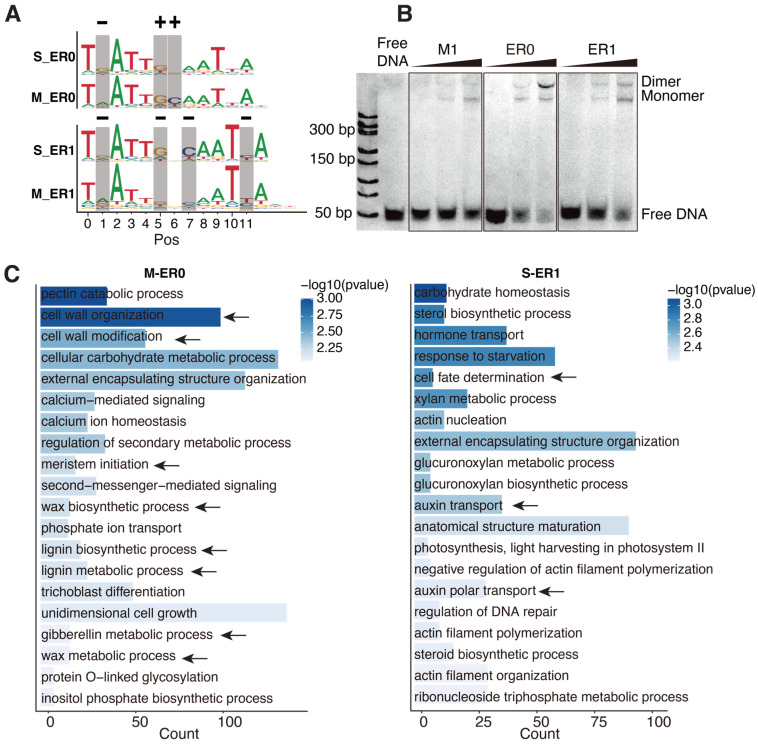
ER0 and ER1 regulate different physiological functions. (**A**) Comparison of the methylated motifs of ER0 and ER1 with their unmethylated counterparts. Cytosine affinity can either increase (“+”) or decrease (“−”) after methylation. (**B**) Electrophoretic mobility shift assay (EMSA) of WOX14 binding to monomer, ER0, and ER1 consensus. The concentrations of WOX14 are increased from left to right (50, 100, and 200 nM). (**C**) The GO enrichment of the target genes of ER0 and ER1. Arrows indicate the GO terms related to the reported functions of AtWOX14 (the development of vascular tissues and plant growth).

We next focused on the high-affinity dimeric configurations, ER0 and ER1, to investigate how their specificity changes in response to DNA methylation. Positional comparison revealed that methylation affected the affinity of cytosines at positions 1, 5, 6, 7, and 11 (Figure 3A). Interestingly, the affinity of cytosines is not universally reduced upon methylation. Whereas the cytosines at pos1 of ER0/1 and pos11 of ER1 show decreased affinities when methylated (most preferred base changed from G to A, or C to T, “−” sign in Figure 3A), the methylation effects on cytosines at the boundaries of the half-sites (pos5/6 of ER0, corresponding to pos5/7 of ER1) are more complex (Figure 3A)—their affinity increases in ER0, but decreases in ER1. This suggests that the effects of methylation on cytosine affinity can depend on the spacing of the TF dimer, which was also previously unrecognized.

We further validated the dimeric binding of ER0 and ER1 to WOX14 using electrophoretic mobility shift assays (EMSAs). Recombinant WOX14 protein was incubated with DNA ligands containing ER0/ER1 consensus sequence and also the consensus sequence of the monomeric motif M1 as a control (Figure 3B). The results show that all ligands can bind WOX14 monomer; however, only ER0 and ER1 ligands can bind dimers of WOX14 at high protein concentrations (Figure 3B). This indicates that ER0 and ER1 are genuine homodimeric CREs of WOX14 and also suggests that the protein-level dimerization of AtWOX14 is weak (no dimeric shift for M1 ligand); a proper CRE on DNA is necessary to facilitate its dimerization. This could explain why the dimeric configuration of AtWOX14 is sensitive to DNA methylation.

To explore the potential biological roles of the ER0 and ER1 CREs, we conducted a Gene Ontology (GO) analysis of their target genes (Figure 3C) using motif matches in the promoter regions. We found that there is almost no overlap between the biological processes regulated by ER0 and ER1. Considering only the reported functions (arrows in Figure 3C) for AtWOX14 [9,10,11,13], genes downstream of ER0 were enriched in biological processes such as cell wall organization, cell wall modification, lignin metabolism and biosynthesis, gibberellin metabolism, and meristem initiation (Figure 3C). In contrast, genes associated with ER1 were predominantly involved in cell fate determination and auxin transport. Therefore, DNA methylation could facilitate AtWOX14 to utilize different dimeric binding modes (ER0 vs. ER1) and regulate distinct physiological processes.

### 2.4. Methylation Effects on Genome-Wide Binding Targets of AtWOX14

**Figure 4:**
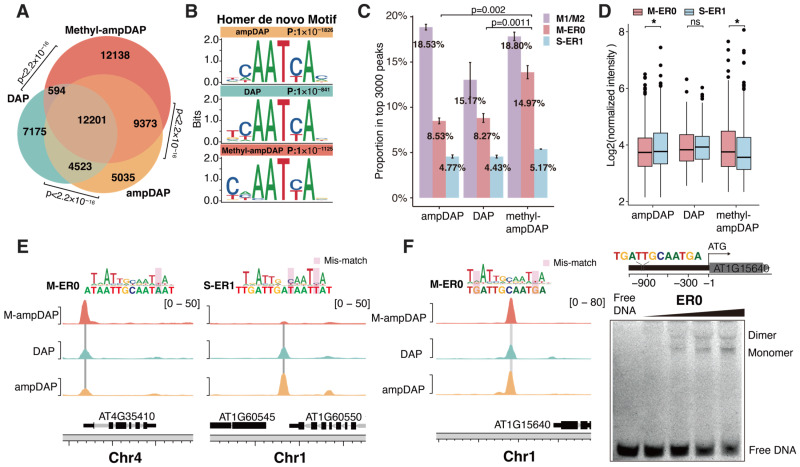
The genome-wide binding of AtWOX14 in the presence and absence of methylation. (**A**) Venn diagram comparing peaks identified in DAP, ampDAP, and Methyl-ampDAP; significant overlaps were observed between each pair of peak sets. *p* values from Fisher’s Exact Test. (**B**) PWM models of the most enriched motifs identified from DAP, ampDAP, and Methyl-ampDAP peaks using Homer. (**C**) Proportions of the top 3000 peaks that contain each motif. The proportion of ER0 in Methyl-ampDAP is significantly higher than in ampDAP and DAP. *p* values from *t*-tests. (**D**) Intensities of peaks that contain ER0 or ER1 motif. *p* values from *t*-tests. “*” indicates *p* < 0.05 and “ns” indicates non-significant difference. (**E**) Normalized coverage tracks of Methyl-ampDAP, DAP, and ampDAP near the ER0/ER1 target sites. The ER0/ER1 CRE sequences are displayed above, with their genomic positions marked by gray vertical lines and mismatches to the consensus marked by red rectangles. (**F**) The EMSA (right) shows the dimeric binding of the WOX14 protein to the ER0 CRE sequence within the promoter of AT1G15840 (left). In the EMSA, the concentrations of WOX14 are 25, 50, 100, and 200 nM, from left to right.

To explore the impact of methylation on the genomic binding sites of AtWOX14, we generated native (DAP-seq), unmethylated (ampDAP-seq), and fully methylated (Methyl-ampDAP-seq) DAP-seq libraries. Similarly to methyl-SELEX, methyl-ampDAP was performed by amplifying genomic DNA with 5mCs instead of Cs during PCR. The Venn diagram (Figure 4A) revealed that the DAP and ampDAP libraries are more similar to each other, while methyl-ampDAP identified many more (12,138) unique peaks. The similarity between DAP and ampDAP can potentially be explained by the relative scarcity of CHH methylation in the genome.

Motif discovery of the DAP-seq libraries using Homer [37] has confirmed that the peak sites of all libraries contained the monomer motif of WOX14 (Figure 4B). These motifs are consistent with those derived from SELEX (Figure 2A, B). From the DAP libraries, we also confirmed that ER0 CREs were preferentially bound under methylated conditions (Figure 4C–E) because (1) there are more peaks containing ER0 in Methyl-ampDAP than DAP/ampDAP libraries (Figure 4C) and (2) the intensity of ER0 peaks is also higher than ER1 peaks in the Methyl-ampDAP but not in the DAP/ampDAP libraries (Figure 4D). In contrast to ER1, while the number of peaks containing ER0 is similar across all the DAP libraries (Figure 4C), the intensity of ER0 peaks is weaker than ER1 peaks only in the ampDAP library (Figure 4D). We next examined the individual binding sites of AtWOX14 and found that the ER0 site near AT4G35410 showed the highest occupancy upon methylation (Figure 4E, left), whereas the ER1 site upstream of AT1G60545 showed the highest occupancy in the absence of methylation (Figure 4E, right). The ER0 site within the promoter of AT1G15640 harbors a sequence almost the same as the consensus of the ER0 motif (Figure 4F, left). We also validated its capability to bind the AtWOX14 dimer using EMSA (Figure 4F, right).

Overall, the analysis of DAP-seq libraries also confirmed that upon methylation, AtWOX14 switched its preferred dimeric mode from ER1 to ER0.

## 3. Discussion

**Figure 5:**
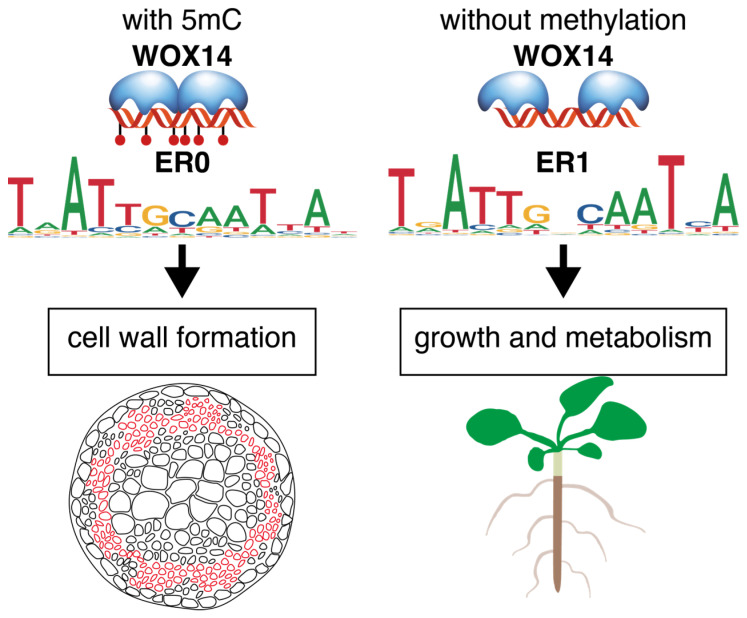
Cytosine methylation changes the preferred dimeric configuration and regulatory function of AtWOX14.

This study performed SELEX and DAP-seq to investigate the impact of cytosine methylation on the specificity and genome-wide binding sites of AtWOX14. The most interesting finding is that the preferred dimeric configuration shifts from ER1 to ER0 upon DNA methylation (Figure 4E). ER0 and ER1 were also found to regulate target genes with different functions (Figure 3C). Therefore, it is reasonable to hypothesize that when genome-wide methylation level is low, AtWOX14 will regulate cell differentiation and cell fate determination processes that are related to growth and metabolism [9,10] (Figure 5). However, as genome-wide methylation level increases, such as when the level of methyl-transferase DRM increases or when the RdDM pathway is activated [38], AtWOX14 may shift its binding preference from ER1 to ER0 to regulate gibberellin synthesis and accumulation, thereby promoting the lignification of vascular cells [13] (Figure 3C and Figure 5). Consequently, the methylation level may serve as a switch, toggling between different downstream regulatory networks of AtWOX14.

This mechanism can potentially be extrapolated to other TFs that bind as homodimers but not protein-level homodimers. This is because, in general, protein-level dimerization of TFs depends on amino acids outside the DNA-binding domain (DBD) [39], which tends to be far away from the DNA and thus unlikely affected by DNA methylations [40]. However, if the protein-level interaction between TF monomers is weak, then the homodimeric binding can be heavily mediated by the DNA scaffold. In this case, it is conceivable that the chemical modifications on DNA could exert more prominent effects on the preferred dimeric configurations. Notably, dimeric binding mediated by the DNA scaffold is not a rare case but represents a prevalent phenomenon, because a high-throughput study has revealed that many TFs only form very few contacts between the two monomers when docked to the high-affinity dimeric CRE sequences. The limited number of protein-level contacts is not sufficient to stabilize a protein-level TF dimer. The solved TF-DNA structures also suggest that TF dimers can form purely dependent on DNA, with two monomers spaced at >10 Å (e.g., PDB: 1HJB) [41,42]. Dimeric binding without direct contacts can be induced by the allosteric effect [43], or if a TF alters the DNA shape upon binding.

When addressing the methylation effects on a TF’s binding affinity and specificity, the previous work mainly relied on comparing the DAP and ampDAP libraries. This approach artfully made use of the sporadic 5mCs present in the native genome, to interrogate how methylation changes the affinity of each C in a known motif model of the TF [22]. However, this method is limited by its inability to de novo build binding models in the presence of 5mC. Also, because the non-CG methylation sites on the genome are relatively sparse [44], the methylation effect may be overfitted with limited data points. In this work, we employed the recently developed Methyl-SELEX method [30] to generate input DNA ligands with 100% 5mC and extrapolated the protocol to set up Methyl-ampDAP. These methods will largely facilitate our understanding of the methylation effects of TF binding.

## 4. Materials and Methods

### 4.1. Cloning and Protein Expression

The full-length CDS sequence of WOX14 was cloned into the bacterial expression vector pETG20A-His using the In-Fusion Kit (Vazyme, C112, Nanjing, China). This resulted in the generation of an N-terminal His-tagged fusion protein construct of WOX14. The construct was then transformed into Rosetta 2 (DE3) pLysS strains and first cultured at 37 °C under shaking conditions (800 rpm) (Miulab, MIX-1500, Hangzhou, China) for ~6 h to allow proliferation. The culture was then shifted to 17 °C and incubated overnight on shaker (Miulab, MIX-1500, Hangzhou, China) at 800 rpm to induce the expression of recombinant WOX14 protein overnight at 17 °C under shaking conditions (800 rpm). Expression of the protein was confirmed by Western blot. After an overnight incubation, the bacteria were collected by centrifugation (Eppendorf, 5810R, Hamburg, Germany) at 10,000 rpm for 3 min and resuspended in 150 µL of Buffer A (50 mM Tris (Sangon, A600194, Shanghai, China), 300 mM NaCl (Sangon, A610476, Shanghai, China), 10 mM Imidazole (Sangon, A419472, Shanghai, China), pH 7.5). The bacterial suspension was then frozen at −80 °C overnight. The frozen bacteria were lysed using lysis buffer (0.5 mg/mL lysozyme (Sigma, 1.05281, St. Louis, MO, USA), 2 mg/mL DNase I (Sangon, B618252, Shanghai, China), 1 mM PMSF (Sangon, A610425, Shanghai, China)) at room temperature for 2 h. Following lysis, His-tag Ni Sepharose (Sangon, C600332, Shanghai, China) was added and incubated with the lysate in a shaker (Miulab, MIX-1500, Hangzhou, China) at 800 rpm for 1 h, then washed with Buffer A and Buffer B (Buffer A plus 50 mM Imidazole (Sangon, A419472, Shanghai, China)) using a 10 kD ultra-centrifugal filter (Sigma, UFC8010, St. Louis, MO, USA) to remove non-specifically bound proteins. The purified WOX14 protein was eluted from the beads using Buffer C (Buffer A plus 500 mM Imidazole (Sangon, A419472, Shanghai, China), pH 7.5).

### 4.2. SELEX

SELEX (Systematic Evolution of Ligands by Exponential Enrichment) was performed following previously described protocols [45]. Briefly, the input SELEX DNA library was designed to include a central randomized region of 101 bp and flanking regions that were compatible with Illumina sequencing (TruSeq adapter sequences). Then, 6 µL purified WOX14 proteins were mixed with 6 µL of SELEX DNA library (30 ng/µL), 2 µL His-Tag Beads (Cytiva, 17371222, Uppsala, Sweden), and 30 µL of TCAPT buffer (140 mM KCl (Macklin, C153347871, Shanghai, China), 5 mM NaCl (Sangon, A610476, Shanghai, China), 1 mM MgCl_2_ (Sangon, A610328, Shanghai, China), 3 µM ZnSO_4_ (Sangon, A602906, Shanghai, China), 100 µM EGTA (Macklin, C11524832, Shanghai, China), 10 mM Tris (Sangon, A600194, Shanghai, China), pH 8, 0.1% Tween (Solarbio, Cat#T8220, Beijing, China)) in a shaker (Miulab, ST60-4, Hangzhou, China) at 800 rpm and 25 °C for 1 h to facilitate DNA–protein binding. The DNA–protein complexes were enriched and pulled down using the His-Tag Beads (Cytiva, 17371222, Uppsala, Sweden), followed by removing unbound DNA ligands using the TCAPT buffer on the HydroSpeed plate washer (Tecan, 30190101, Zurich, Switzerland). The complexes were eluted with 30 μL elution buffer (0.1% Tween (Solarbio, Cat#T8220, Beijing, China), 10 mM Tris (Sangon, A600194, Shanghai, China), pH 7.8, 1 mM MgCl_2_ (Sangon, A610328, Shanghai, China)). Subsequently, the bound DNA ligands were amplified for 15~20 cycles using amplification primers (forward primer 5′-CCCTACACGACGCTCTTCC-3′, reverse primer 5′-CAGACGTGTGCTCTTCCG-3′), and the resulting PCR products were used as the input library for the next cycle. After 5 cycles, the enriched DNA ligands were amplified using the PE primers (forward PE primer 5′-AATGATACGGCGACCACCGAGATCTACACTCTTTCCCTACACGACGCTCTTCC-3′, reverse PE primer 5′-CAAGCAGAAGACGGCATACGAGAT-barcode-GTGACTGGAGTTCAGACGTGTGCTCTTCCG-3′) and subjected to Illumina sequencing.

### 4.3. Methyl-SELEX

The Methyl-SELEX process derives from SELEX with the addition of a DNA methylation step [30]. Specifically, the input SELEX DNA library was first amplified by PCR using 5-methyl-dCTP to generate a methylated DNA library. This methylated library was then mixed with 6 µL purified WOX14 protein and 30 µL of TCAPT buffer in a shaker (Miulab, ST60-4, Hangzhou, China) at 800 rpm and 25 °C for 1 h. Methylated DNA ligands bound to WOX14 were separated from the unbound sequences via a washing step and amplified using 5-methyl-dCTP as input for subsequent cycles.

### 4.4. Data Analyses of SELEX

The raw sequencing data generated from the Illumina NovaSeq 6000 platform were first demultiplexed according to the unique i7 index of each sample. In the pre-processing stage, the low-quality reads and PCR duplicates were removed, the adaptors were trimmed, and then the paired-end reads were subsequently merged. To de novo discover the binding motifs of WOX14 from the pre-processed SELEX reads, Autoseed was run with parameters “-40N <Background sequence> <Signal sequence> 1 8 10 0.35-40 100” [33]. The previously described enrichment-based mutual information (E-MI) analysis was used to evaluate the signal strength of transcription factors in the SELEX library [31,32]. To calculate motif enrichments, motif matching analysis was performed using the R package motifmatchr v1.22.0 [46]. The “matchMotifs” function was used to identify motif hits in DNA sequences by comparing subsequences with the position weight matrices (PWMs). We performed motif matching for the SELEX libraries with a *p*-value of 1 × 10^−5^. Additionally, we generated a shuffled version of the SELEX library to serve as a background, and motif matching was also performed on this shuffled library. The final motif enrichments were calculated by dividing the number of motif hits in the SELEX library by that in the shuffled library, providing a measure for the signal strength of each binding motif in the SELEX libraries. Gene Ontology (GO) analyses were conducted using the R package clusterProfiler v4.8.1 [47].

### 4.5. Electrophoretic Mobility Shift Assays

The protein–DNA binding buffer was prepared with 10 mM Tris, 50 mM NaCl, 1 mM MgCl_2_, 4% glycerol, and 0.5 mM EDTA. Double-stranded DNA probes, either containing monomeric or dimeric binding sequences, were synthesized and diluted to a concentration of 50 nM. The probe sequences used in EMSA were as follows:

M1: ATGCTAGCTCCATCTGTATTGATTGTTTATGGCGGTGACGTACT;
ER0: ATGCTAGCTCCATCTGTGATTGCAATCAATGGCGGTGACGTACT;
ER1: ATGCTAGCTCCATCTGTGATTGACAATCAATGGCGGTGACGTACT;
ER0-AT1G15640: AATAAGATGTACCCAATGATTGCAATGATGAGCCCAATGA GTGC.

The purified WOX14 protein was then sequentially diluted and incubated with 30 ng of the DNA probe in a 20 µL protein–DNA binding buffer at 25 °C for 40 min. The resulting reaction mixture was loaded onto a 6% native PAGE gel and run in 0.5× TBE buffer at 4 °C for 90 min at 110 V. The gel was subsequently stained with Gel-Blue (UElandy, S2019L, Suzhou, China) and imaged using a Bio-Rad scanner (Bio-Rad, 1708195EDU, Hercules, CA, USA).

### 4.6. DAP and Data Pre-Processing

The ampDAP, methyl-ampDAP, and DAP protocols followed the previously described workflow [48,49]. Briefly, genomic DNA (gDNA) was extracted from 7-day-old *Arabidopsis* seedlings using the Genomic DNA Extraction Kit (Tiangen, A0903A, Beijing, China) and fragmented using the Covaris S2 sonicator. The fragmented DNA (the average size of the genomic DNA was 150–300 bp), containing natural DNA methylation modification, was then purified with DNA clean beads (Vazyme, N411, Nanjing, China) and ligated with adapters using the ligation kits (Vazyme, N203, N204, Nanjing, China) to prepare the input library for DAP. For the input library of amp-DAP, the gDNA was further amplified with dCTP to obtain completely unmethylated gDNA. For the input library of methyl-amp-DAP, the gDNA was amplified by PCR with 5-methyl-dCTP to generate fully methylated gDNA. The subsequent steps are shared for DAP, ampDAP, and methyl-ampDAP: 6 µL of His-tagged WOX14 protein was mixed with ~200 ng of input DAP library, 2 μL His-Tag Beads (Cytiva, 17371222, Uppsala, Sweden), and 30 µL of TCAPT buffer, and the mixture was incubated in a shaker (Miulab, ST60-4, Hangzhou, China) at 800 rpm and 25 °C for 1 h. The protein-bound DNA was then collected by pulling down the beads and washed using TCAPT buffer on a HydroSpeed plate washer (Tecan, 30190101, Zurich, Switzerland). The collected DNA ligands were eluted with 30 μL elution buffer (0.1% Tween (Solarbio, Cat#T8220, Beijing, China), 10 mM Tris (Sangon, A600194, Shanghai, China), pH 7.8, and 1 mM MgCl_2_ (Sangon, A610328, Shanghai, China)) and amplified using the PE primers and sequenced on Illumina NovaSeq 6000.

Raw reads were demultiplexed, and sequences with low quality or adapters were removed before aligning to the TAIR10 version of the Arabidopsis genome (https://www.arabidopsis.org/download_files/Genes/TAIR10_genome_release/TAIR10_chromosome_files/TAIR10_chr_all.fas.gz (accessed on 8 March 2024)) using BWA v.0.7.17 [50]. Genome annotations were obtained from the Araport11 files (https://www.arabidopsis.org/download/list?dir=Genes%2FAraport11_genome_release (accessed on 7 October 2024)). Peak calling was performed with MACS3 v.3.0.0a7 [51]. Motif discovery was performed using Homer v5.1 (http://homer.ucsd.edu/homer/ (accessed on 8 March 2024)) [37]. BigWig files of normalized read signals were created by the “bamCoverage” program in deepTools v3.5.1 [52]. The genome browser tracks were plotted from the read normalized bigwig files using the R package karyoploteR v1.26.0 [53]. Data analysis and visualization were performed in R v.4.4.0.

### 4.7. Statistical Analysis

Fisher’s exact test (performed using the “fisher.test” function from the stats package in R v4.4.0) was used to evaluate the overlaps between each pair of peak sets. Student’s *t*-test (conducted with the “t.test” function from the stats package in R v4.4.0) was employed to analyze changes in the proportion of the top 3000 peaks containing each motif and to compare the intensities of ER0 and ER1 peaks in different DAP-seq libraries. All statistical tests were two-sided, and *p*-values ≤ 0.05 were considered statistically significant. Asterisks (*) indicate *p* ≤ 0.05, while “ns” denotes *p* > 0.05. All analyses were performed using R v4.4.0.

## 5. Conclusions

Our study comprehensively analyzed the DNA-binding specificity and genome-wide binding targets of AtWOX14 and revealed the impact of DNA methylation on TF-DNA interactions. We found that AtWOX14 preferentially binds DNA in a dimeric configuration both in the presence and absence of methylation. The methylation effect on a cytosine’s affinity to AtWOX14 is position-dependent. Interestingly, while 5mC overall represses DNA binding of AtWOX14 monomers, it facilitates the binding of AtWOX14 dimers. The preferred dimeric configuration also changed from ER1 to ER0 upon methylation, highlighting the importance of the DNA scaffold in determining the dimeric binding of AtWOX14 and indicating a previously unrecognized mechanism by which plants can use the same TF to regulate different downstream regulatory pathways in response to the genome-wide DNA methylation level.

## Data Availability

The raw sequence data reported in this paper have been deposited in the Genome Sequence Archive (Genomics, Proteomics & Bioinformatics 2021) in China National Center for Bioinformation under the accession number PRJCA032646.

## Supplementary Materials

The following supporting information can be downloaded at: https://www.mdpi.com/article/10.3390/ijms26020763/s1.
