# Supplementary material for: Cytosine Methylation Changes the Preferred Cis-Regulatory Configuration of Arabidopsis WUSCHEL-Related Homeobox 14
Source: Int J Mol Sci. 2025 Jan 17;26(2):763. doi: 10.3390/ijms26020763 (PMC11765556; doi:10.3390/ijms26020763)
Supplement: Supplementary file 1 [file ijms-26-00763-s001.zip › ijms-3378305-supplementary.pdf]

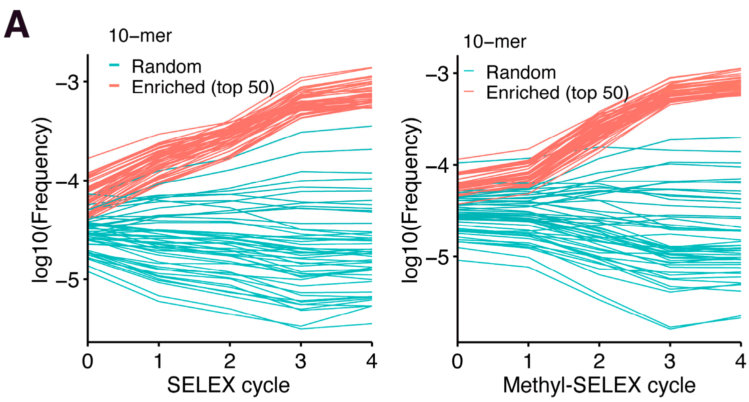

Figure S1. (A) The frequencies of subsequence (10-mers) after taking the logarithm in each cycle of WOX14 SELEX and Methyl-SELEX. In addition to the bound sequences (top 50 enriched sequences, red), 50 randomly selected sequences (blue) were also visualized.
